# Reversed Senescence of Retinal Pigment Epithelial Cell by Coculture With Embryonic Stem Cell via the TGFβ and PI3K Pathways

**DOI:** 10.3389/fcell.2020.588050

**Published:** 2020-11-26

**Authors:** Shoubi Wang, Yurun Liu, Ying Liu, Chaoyang Li, Qi Wan, Liu Yang, Yaru Su, Yaqi Cheng, Chang Liu, Xiaoran Wang, Zhichong Wang

**Affiliations:** State Key Laboratory of Ophthalmology, Zhongshan Ophthalmic Center, Sun Yat-sen University, Guangzhou, China

**Keywords:** embryonic stem cell, age-related macular degeneration, retinal pigment epithelium cell, reversing cellular senescence, regulatory mechanism

## Abstract

Retinal pigment epithelium (RPE) cellular senescence is an important etiology of age-related macular degeneration (AMD). Aging interventions based on the application of stem cells to delay cellular senescence have shown good prospects in the treatment of age-related diseases. This study aimed to investigate the potential of the embryonic stem cells (ESCs) to reverse the senescence of RPE cells and to elucidate its regulatory mechanism. The hydrogen peroxide (H_2_O_2_)-mediated premature and natural passage-mediated replicative senescent RPE cells were directly cocultured with ESCs. The results showed that the proliferative capacity of premature and replicative senescent RPE cells was increased, while the positive rate of senescence-associated galactosidase (SA-β-GAL) staining and levels of reactive oxygen species (ROS) and mitochondrial membrane potential (MMP) were decreased. The positive regulatory factors of cellular senescence (p53, p21^WAF1/CIP1^, p16^INK4a^) were downregulated, while the negative regulatory factors of cellular senescence (Cyclin A2, Cyclin B1, Cyclin D1) were upregulated. Furthermore, replicative senescent RPE cells entered the S and G_2_/M phases from the G_0_/G_1_ phase. TGFβ (TGFB1, SMAD3, ID1, ID3) and PI3K (PIK3CG, PDK1, PLK1) pathway-related genes were upregulated in premature and replicative senescent RPE cells after ESCs application, respectively. We further treated ESCs-cocultured premature and replicative senescent RPE cells with SB531542 and LY294002 to inhibit the TGFβ and PI3K pathways, respectively, and found that p53, p21^WAF1/CIP1^ and p16^INK4a^ were upregulated, while Cyclin A2, Cyclin B1, Cyclin D1, TGFβ, and PI3K pathway-related genes were downregulated, accompanied by decreased proliferation and cell cycle transition and increased positive rates of SA-β-GAL staining and levels of ROS and MMP. In conclusion, we demonstrated that ESCs can effectively reverse the senescence of premature and replicative senescent RPE cells by a direct coculture way, which may be achieved by upregulating the TGFβ and PI3K pathways, respectively, providing a basis for establishing a new therapeutic option for AMD.

## Introduction

Age-related macular degeneration (AMD) is the major cause of blindness around the world (Mitchell et al., 2018), and there is currently no effective treatment for AMD. In recent years, the number of AMD patients has increased year by year and is estimated to reach 288 million in 2040 (Wong et al., 2014), resulting in a heavy social burden. Intravitreal injection of anti-vascular endothelial growth factor (VEGF) drugs is currently the most effective treatment for neovascular AMD, but it is expensive and is easy to relapse after drug withdrawal. There is currently no effective treatment for dry AMD. Although stem cells can be used to differentiate into functional retinal pigment epithelium (RPE) cells, there are some problems, such as low differentiation efficiency, tumorigenicity and unknown safety issues (Mandai et al., 2017), which limits their clinical application. Hence, how to use stem cells safely and effectively in the treatment of AMD is an urgent problem to be solved.

Retinal pigment epithelium cellular senescence is one of the main factors in the development of AMD (Wang et al., 2019). Hence, prevention and reversal of RPE cellular senescence may be a therapeutic strategy for AMD. Antioxidant drugs, such as fullerenol and humanin, have been applied to reduce oxidative stress and DNA damage in RPE cells (Zhuge et al., 2014; Sreekumar et al., 2016), thereby delaying RPE cellular senescence. However, almost all drugs have off-target and bystander effects. In addition, delaying cellular senescence cannot clear existing senescent cells, so the progression of age-related diseases cannot be stopped by antioxidant drugs. Therefore, finding a method to effectively reverse RPE cellular senescence may provide new insight for AMD treatment.

The embryonic microenvironment can reverse somatic cellular senescence. The cloning of Dolly the sheep is a good example. A mature mammary gland cell can be reprogrammed into a stem cell under the influence of the embryonic microenvironment and can ultimately result in the cloning of a new individual. However, this embryonic microenvironment cannot be used clinically. The embryonic stem cells (ESCs) can mimic the role of the embryonic microenvironment *in vitro*. Studies have shown that ESC-conditioned medium can enhance the survival of bone marrow precursor cells (Guo et al., 2006) and reduce the aging phenotype of senescent skin fibroblasts (Bae et al., 2016). We previously demonstrated that the ESC-conditioned medium could promote the proliferation of corneal epithelial and endothelial cells *in vitro* (Liu et al., 2010; Lu et al., 2010), and showed that ESCs could maintain stemness in corneal epithelial cells by the transwell indirect coculture and the cell-contact-cell direct coculture ways, which was achieved by regulating the telomerase pathway (Zhou et al., 2011), with telomerase shortening being an important indicator of cellular senescence. In addition, we also demonstrated that the ESCs can reverse the malignant phenotype of tumors by a direct coculture way and promote the proliferation of normal skin tissues adjacent to tumors (Liu et al., 2019). Therefore, ESCs may have the potential to reverse the senescence of RPE cells.

On this basis, we applied ESCs to hydrogen peroxide (H_2_O_2_)-mediated premature senescent RPE cells and natural passage-mediated replicative senescent RPE cells by a direct coculture way in this study. Cellular senescence was dynamically assessed according to the changes in the proliferative capacity of RPE cells, senescence-associated galactosidase (SA-β-GAL) staining activity, cell cycle distribution, levels of reactive oxygen species (ROS) and mitochondrial membrane potential (MMP), and expression of cellular senescence markers (p53, p21^WAF1/CIP1^, p16^INK4a^, Cyclin A2, Cyclin B1, and Cyclin D1). The mechanism was further clarified by transcriptome sequencing (RNA-seq), RT-PCR, western blotting and immunofluorescence, aiming to provide a new therapeutic option for stem cell therapy for AMD.

## Materials and Methods

### Cell Culture

Human primary RPE cells were obtained from the eyeballs of donors aged 20–40 who died unexpectedly without eye diseases from the Eye Bank of Guangdong Province (Zhongshan Ophthalmic Center, Sun Yat-sen University) in line with the principles of the Declaration of Helsinki for research involving human tissues. Approval was granted by the Ethics Committee of Zhongshan Ophthalmic Center, Sun Yat-sen University (Ethics approval number: 2020KYPJ031). The cell sampling method was performed as described previously (Rabin et al., 2013). RPE cells were cultured in DMEM/F-12 (Corning, United States) medium containing 1% penicillin-streptomycin (Gibco, Australia) and 10% fetal bovine serum (Gibco) and passaged at a density of 6000/cm^2^ every 2–3 days. Mouse ESC-E14s were provided by Prof. Andy Peng Xiang from Sun Yat-sen University, China (Chen et al., 2006). Then, we used green fluorescent protein to label this cell line to construct the ESC-GFP cell line (Zhou et al., 2014). The ESCs mentioned below are referred to as ESC-E14s-GFP cells. ESCs were cultured as described previously (Liu et al., 2019) and passaged at a density of 1 × 10^4^/cm^2^ every 2–3 days. All cells were cultured in an incubator containing 5% CO_2_ at 37°C.

### Establishment of the Cellular Senescence Model and Coculture System

Retinal pigment epithelium cells from passages 4 to 6 were used in the premature senescence model. RPE cells were treated with 0, 100, 200, 300, 400, and 500 μM H_2_O_2_ in serum-free medium for 4 h and then cultured in complete medium for another 44 h. Next, these cells were collected for cell proliferation and SA-β-GAL staining detection to determine the optimal H_2_O_2_ concentration. After determining the optimal H_2_O_2_ concentration, RPE cells were divided into the following groups: (1) PR group: RPE cells cultured in serum-free medium for 4 h and then cultured in complete medium for another 44 h; (2) PRH group: RPE cells cultured in serum-free medium containing 400 μM H_2_O_2_ for 4 h and then cultured in complete medium for another 44 h; (3) PRHE group: RPE cells cultured in serum-free medium containing 400 μM H_2_O_2_ for 4 h and then directly cocultured with ESCs at a 1:2 ratio in complete medium for another 44 h; and (4) PRHE-SB group: RPE cells cultured in serum-free medium containing 400 μM H_2_O_2_ for 4 h and then directly cocultured with ESCs at a 1:2 ratio in complete medium containing 10 μM SB431542 (MedChemExpress, United States) for another 44 h. RPE cells from passages 8 to 10 were used in the replicative senescence model. RPE cells were divided into the following groups: (1) RR group: RPE cells cultured in complete medium for 48 h; (2) RRE group: RPE cells directly cocultured with ESCs at a 1:2 ratio in completed medium for 48 h; and (3) RRE-LY group: RPE cells directly cocultured with ESCs at a 1:2 ratio in complete medium containing 10 μM LY294002 (MedChemExpress) for 48 h.

### Cell Labeling and Sorting

Before coculture, RPE cells were labeled with 5 μl Vybrant^®^ DiD staining solution (Invitrogen, United States) corresponding to 1 × 10^6^ cells. Finally, RPE cells cocultured with ESCs were isolated by a flow cytometer (BD Bioscience, United States).

### Cell Counting Kit 8 (CCK-8) Cell Proliferation Assay

When determining the optimal concentration of H_2_O_2_, RPE cells were plated in a 96-well plate at 1800 cells/well for 24 h, cultured with 0–500 μM H_2_O_2_ for 4 h, and then cultured in complete medium for another 44 h. Cells from each group were collected and plated in a 96-well plate at 300 cells/well. After 24 h, 10 μl CCK-8 (Dojindo, Japan) solution was added to each well and incubated for 3 h with 5% CO_2_ at 37°C. The optical density was measured by a microplate reader (BioTek, United States) at 450 nm. The CCK-8 assay was performed continuously for 7 days.

### SA-β-GAL Staining Activity

Retinal pigment epithelium cells of each group were collected and plated into a 6-well plate at 1 × 10^6^ cells/well overnight. According to the instructions (Cell Signaling Technology, United States), cells in each well were fixed with 1 ml of 1 × fixative solution for 10–15 min and washed twice with PBS. One milliliter of 1 × β-galactosidase staining solution was added to each well. Cells were incubated in a drying oven at 37°C for 12–14 h and washed twice with PBS. Then, 70% glycerin was added to each well. The SA-β-GAL^+^ cells with blue perinuclear staining were observed by a microscope (Leica, Germany). At least three fields were randomly selected to calculate the positive staining rate.

### ROS Assay

According to the manufacturer’s instructions (Abcam, United Kingdom), the 2′,7′-dichlorofluorescein diacetate (DCFDA) solution was diluted 1000 times with PBS. At least 2 × 10^5^ cells of each group were incubated with 500 μl DCFDA-PBS working solution for 30 min with 5% CO_2_ at 37°C. The mean fluorescence intensity (Ex485 nm/Em535 nm) was measured by a flow cytometer (BD LSRFortessa, United States).

### MMP Assay

Cells from each group were plated into a 96-well plate with a black bottom at a density of 1 × 10^4^ cells/well. On the second day, cells were incubated with 100 μl of 200 nM TMRE (Cell Signaling Technology) for 30 min in a dark incubator with 5% CO_2_ at 37°C and then washed twice with PBS. The mean fluorescence intensity (Ex550 nm/Em580 nm) was measured by a microplate reader (BioTek).

### Cell Cycle Analysis

A total of 1 × 10^6^ cells from each group were fixed with ice-cold 70% ethanol and placed at 4°C overnight. The next day, after washing with PBS, the cells were incubated with 0.5 ml FxCycle^TM^ PI/RNase (Invitrogen) for 15–30 min at room temperature. The cell cycle distribution (Ex488 nm) was detected by a flow cytometer (BD LSRFortessa).

### RT-PCR

Total RNA was extracted using an RNeasy mini kit (QIAGEN, Germany). The concentration of total RNA was measured using a NanoDrop 1000^TM^ spectrophotometer (Thermo Fisher Scientific, United States). Reverse transcription was performed using the SYBR PrimeScript^TM^ Master Mix kit (Takara, Japan). PCR was performed using the SYBR Premix Ex Taq Kit (Takara). The mRNA expression was measured by a LightCycler 480 (Roche, Switzerland). GAPDH was used as an internal reference. The primer sequences are shown in Table 1.

**TABLE 1:** Primer sequences for RT-PCR analysis.

Genes	Primer sequences
p53	Forward: 5′-GGTCCAGATGAAGCTCCCAGA-3′ Reverse: 5′-AGGGACAGAAGATGACAGGGG-3′
p21^WAF1/CIP1^	Forward: 5′-GTCTTGTACCCTTGTGCCTCG-3′ Reverse: 5′-GCGGATTAGGGCTTCCTCTTG-3′
p16^*INK4a*^	Forward: 5′-GTGGGTTTGTAGAAGCAGGCA-3′ Reverse: 5′-ATCCCCAGGCATCTTTTGCAC-3′
Cyclin A2	Forward: 5′-TGCTGGAGCTGCCTTTCATTT-3′ Reverse: 5′-GCTGTGGTGCTTTGAGGTAGG-3′
Cyclin B1	Forward: 5′-GATCGCCCTGGAAACGCAT-3′ Reverse: 5′-CACTGCTCCCTCCTTATTGGC-3′
Cyclin D1	Forward: 5′-GCTGTGCATCTACACCGACAA-3′ Reverse: 5′-ATGAAATCGTGCGGGGTCATT-3′
TGFB1	Forward: 5′-TCCGTGGGATACTGAGACACC-3′ Reverse: 5′-TCTCCCGGCAAAAGGTAGGAG-3′
SMAD3	Forward: 5′-CAAGTATGGTAGGGGAGGGCA-3′ Reverse: 5′-TGGGTTTGCTCGTGTGTTTCA-3′
ID1	Forward: 5′-GTTACTCACGCCTCAAGGAGC-3′ Reverse: 5′-ATGTAGTCGATGACGTGCTGG-3′
ID3	Forward: 5′-GAGAGGCACTCAGCTTAGCC-3′ Reverse: 5′-TCCTTTTGTCGTTGGAGATGAC-3′
PIK3CG	Forward: 5′-AACACCGACCTCACAGTTTTT-3′ Reverse: 5′-CTCAAGCCACACATTCCACAG-3′
PDK1	Forward: 5′-AGAGGGTTACGGGACAGATGC-3′ Reverse: 5′-GTCTTTGGGTTCTCTGCTGGG-3′
PLK1	Forward: 5′-GGACTGGCAACCAAAGTCGAA-3′ Reverse: 5′-CACCTCGAAACTGTGCCCTTT-3′
GAPDH	Forward: 5′-AAAATCAAGTGGGGCGATGCT-3′ Reverse: 5′-GGTTCACACCCATGACGAACA-3′

### Western Blotting

Cells from each group were collected, and 200 μl of 1× sodium dodecyl sulfate (SDS) was added to 1 × 10^6^ cells to lyse the cells on ice for 30 min. The cell lysates were boiled for 10 min on a dry thermostat (Essenscien, United States) and centrifuged at 14000 rpm for 20 min. Finally, the supernatants were extracted from the cell lysis solutions and stored at −80°C for later use. Protein quantification was performed using a BCA Protein Assay Kit (Bio-Rad, Canada). A 30 μg sample from each group was loaded in a 10% SDS-PAGE gel. After electrophoresis for 1.5 h, the proteins on the gel were transferred to a PVDF membrane at 300 mA for 3 h. Then, the membrane was blocked with 5% non-fat dry milk in TBST (Tris-buffered saline with 0.1% Tween-20) for 2 h and incubated with primary antibodies at 4°C overnight. After washing three times with TBST, the membrane was incubated with horseradish peroxidase-linked anti-mouse (1:5000, Sigma) and horseradish peroxidase-linked anti-rabbit (1:5000, Sigma) secondary antibodies for 1.5 h at room temperature. After washing three times with TBST again, the intensity of the protein bands was detected by a ChemiDoc MP imaging system (Bio-Rad, United States) using an ECL substrate (Thermo Fisher Scientific, United States). Information on the primary antibodies is shown in Supplementary Table 1. Quantification results are shown in Supplementary Figure 1.

### Immunofluorescence

Cells of each group were collected and seeded in a 6-well plate with coverslips at a density of 5 × 10^4^ cells per well. After attaching to the plate, the cells were fixed with 4% paraformaldehyde, permeabilized with 0.3% Triton X-100, and blocked with goat serum at room temperature for 20 min. Then, the cells were incubated with the primary antibodies at 4°C overnight, after which they were incubated with Alexa Fluor 594 donkey anti-mouse lgG secondary antibody or Alexa Fluor 594 donkey anti-rabbit lgG secondary antibody (1:100, Invitrogen) at room temperature for 1 h. The nuclei were stained with Hoechst 33258 (1:2000, Invitrogen) for 10 min. Finally, an anti-fluorescence quenching agent (Bosterbio, United States) was used to prevent fluorescence quenching. PBS was used for washing three times after every step. Immunofluorescence images were taken by a laser scanning confocal microscope (LSM 800; Carl Zeiss, Germany). Cells incubated with PBS instead of primary antibody were used as negative controls. Information on the primary antibodies is shown in Supplementary Table 1.

### RNA-Seq

RNA samples from the PR, PRH, PRHE, RR, and RRE groups were prepared (*n* = 3). RNA-seq was performed using Illumina platform and analyzed by Annoroad Gene Technology Co., Ltd. (Beijing, China). DESeq 2 was used for gene differential expression analysis. The differentially expressed genes of | log2 Fold change| ≥ 1 and *q* < 0.05 were selected as significant differentially expressed genes. The hypergeometric test was used for Kyoto Encyclopedia of Genes and Genomes (KEGG) pathway analysis.

### Statistical Analysis

The statistical analyses were performed by GraphPad Prism 7.0 software. Differences between two groups were analyzed using the two-tailed unpaired Student’s *t*-test, and One-way ANOVA or Two-way ANOVA were used for comparing more than two groups. All data are presented as the mean ± standard deviation (SD). *P*-values < 0.05 were considered statistically significant.

## Results

### Identification of RPE Cells

**FIGURE 1:**
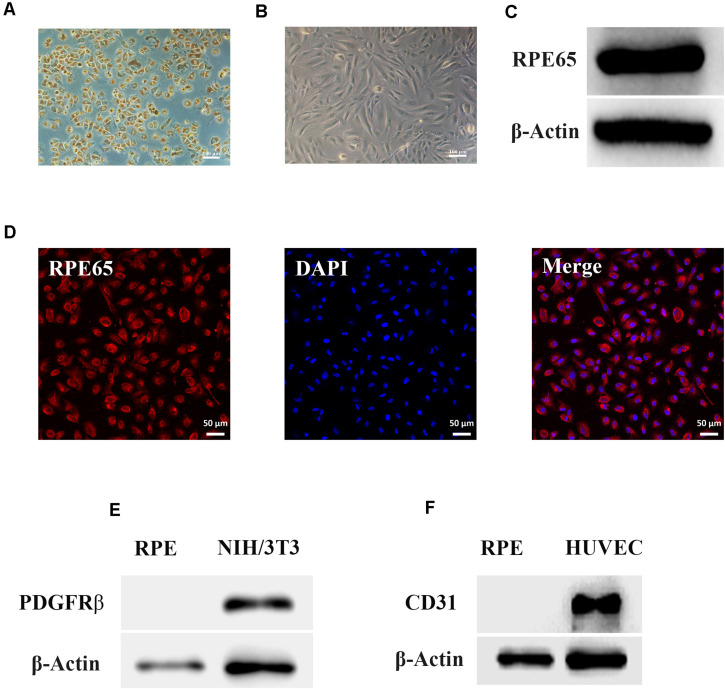
Identification of retinal pigment epithelium (RPE) cells. **(A)** The morphology of RPE cells from passage 0 by phase contrast microscopy. Scale bar, 100 μm. **(B)** The morphology of RPE cells from passage 4 by phase contrast microscopy. Scale bar, 100 μm. **(C)** Western blots of RPE65 in RPE cells. β-Actin served as the internal control. **(D)** Immunofluorescence assays of RPE65 in RPE cells. Scale bar, 50 μm. **(E)** Western blots of PDGFRβ in RPE cells. NIH/3T3 cell was used as the positive control. β-Actin served as the internal control. **(F)** Western blots of CD31 in RPE cells. Human umbilical vein endothelial cell (HUVEC) was used as the positive control. β-Actin served as the internal control.

The morphology of RPE cells is shown in Figures 1A,B. RPE cells in passage 0 had a paving stone morphology and contained a large amount of black pigment (Figure 1A). With increasing passage times, RPE cells gradually became spindle-shaped, and the intracellular pigment decreased (Figure 1B). The results of western blotting (Figure 1C) and immunofluorescence (Figure 1D) showed that the RPE-specific marker RPE65 was expressed in these cells, and other markers for retinal vascular cells, including PDGFRβ and CD31, were not expressed in human RPE cells (Figures 1E,F), indicating the pure RPE cells were applied in this study.

### Establishment of RPE Premature and Replicative Senescence Cellular Models

**FIGURE 2:**
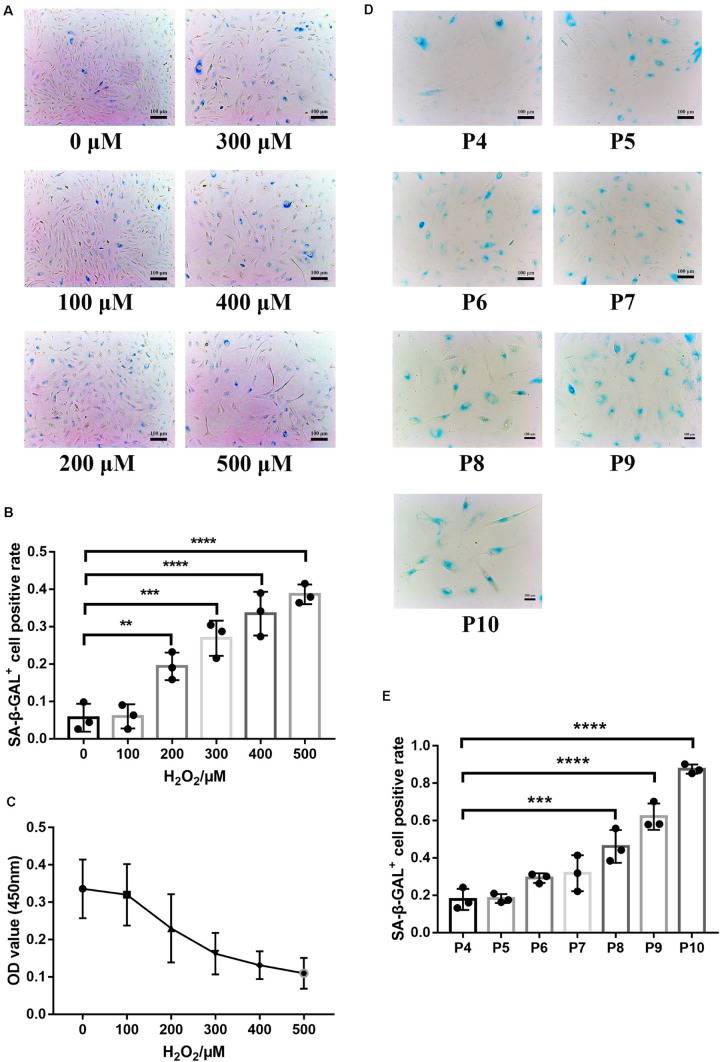
Establishment of RPE premature and replicative senescence cellular models. **(A)** Senescence-associated galactosidase (SA-β-GAL) activity in RPE cells treated with different concentrations of hydrogen peroxide (H_2_O_2_), as indicated by phase contrast microscopy. Scale bar, 100 μm. **(B)** Results from the quantification of SA-β-GAL^+^ cells shown in **(A)**. SA-β-GAL^+^ cells in 4 random fields were scored (*n* = 3 biological repeats). The results are expressed as the percentage of stained cells. **(C)** Proliferation of RPE cells treated with different concentrations of H_2_O_2_, as assessed by a Cell Counting Kit 8 (CCK-8) proliferation assay (*n* = 6 biological repeats). **(D)** SA-β-GAL activity in RPE cells from passages 4 to 10, as indicated by phase contrast microscopy. Scale bar, 100 μm. **(E)** Results from the quantification of SA-β-GAL^+^ cells shown in D. SA-β-GAL^+^ cells in 3 random fields were scored (*n* = 3 biological repeats). The results are expressed as the percentage of stained cells. Data are presented as the mean ± SD. ^∗∗^*P* < 0.01; ^∗∗∗^*P* < 0.001; ^****^P < 0.0001.

SA-β-GAL is the most commonly used indicator of cellular senescence (Piechota et al., 2016). The commonly used models of cellular senescence include (1) stress-mediated premature senescence, which is triggered or accelerated by external factors, independent of telomere shortening, and (2) natural passage-mediated replicative senescence, which represents the limitation of cell proliferation *in vitro* due to telomere shortening (Kida and Goligorsky, 2016; de Magalhaes and Passos, 2018). The retina is vulnerable to oxidative stress because it is rich in mitochondria and contains easily oxidized polyunsaturated fatty acids (PUFAs) (Blasiak et al., 2016). RPE cells are in a chronic oxidative stress state under long-term exposure to light and sustained oxidative stress can lead to DNA damage and a series of cellular senescence reactions (Marazita et al., 2016; Felszeghy et al., 2019; Kaarniranta et al., 2019), indicating that oxidative stress is one of the pathogenic factors of AMD. Hence, oxidative stress was used in this study to establish a model of premature senescence of RPE cells. The application of premature senescent and replicative senescent RPE cells as experimental cells can better reflect the comprehensive role of the ESCs in reversing RPE cellular senescence. In premature senescent RPE cells, the positive rate of SA-β-GAL staining (Figures 2A,B) and proliferation capacity (Figure 2C) were positively and negatively correlated with the H_2_O_2_ concentration, respectively. To ensure that the RPE cells have a certain SA-β-GAL positive staining rate to successfully represent cellular senescence and have a certain proliferative capacity for adequate cell collection, 400 μM H_2_O_2_ was selected as the final experimental concentration. As shown in Figures 2D,E, the positive rate of SA-β-GAL staining gradually increased with increasing cell passages, and there was a significant difference from passage 8. Finally, we selected RPE cells from passages 8 to 10 to represent replicative senescent cells.

### The Cocultured ESCs Increased Proliferative Capacity of Premature and Replicative Senescent RPE Cells

**FIGURE 3:**
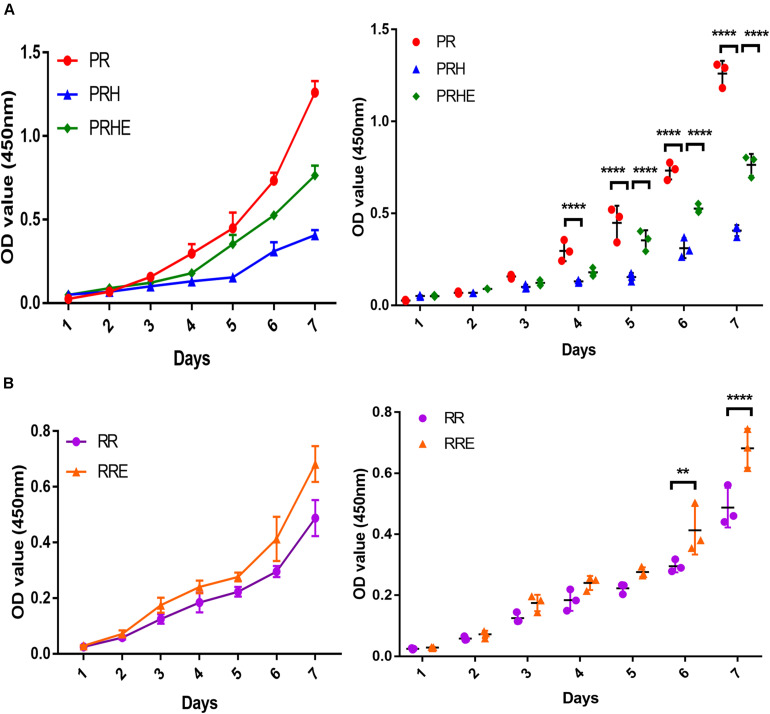
The cocultured embryonic stem cells (ESCs) increased the proliferative capacity of premature and replicative senescent RPE cells. **(A)** Proliferation of the PR, PRH, and PRHE groups, as assessed by a CCK-8 proliferation assay (*n* = 3 biological repeats). **(B)** Proliferation of the RR and RRE groups, as assessed by a CCK-8 proliferation assay (*n* = 3 biological repeats). Data are presented as the mean ± SD. ^∗∗^*P* < 0.01; ^****^P < 0.0001. PR: RPE cells of the control group from passages 4 to 6; PRH: RPE cells from passages 4 to 6 treated with 400 μM H_2_O_2_; PRHE: RPE cells from passages 4 to 6 treated with 400 μM H_2_O_2_ and then cocultured with ESCs. RR: RPE cells of the control group from passages 8 to 10; RRE: RPE cells from passages 8 to 10 cocultured with ESCs.

The CCK-8 assay results showed the proliferation ability and cell growth curve of each group of cells. As shown in Figure 3A, the proliferative capacity of the PRH group was significantly lower than that of the PR group. However, the proliferative capacity of the PRHE (Figure 3A) and RRE (Figure 3B) groups was significantly higher than that of the PRH and RR groups, indicating that the ESCs can improve the proliferative capacity of premature and replicative senescent RPE cells by a direct coculture way.

### The Cocultured ESCs Promoted G_1_/S Phase Transition in Replicative Senescent RPE Cells

**FIGURE 4:**
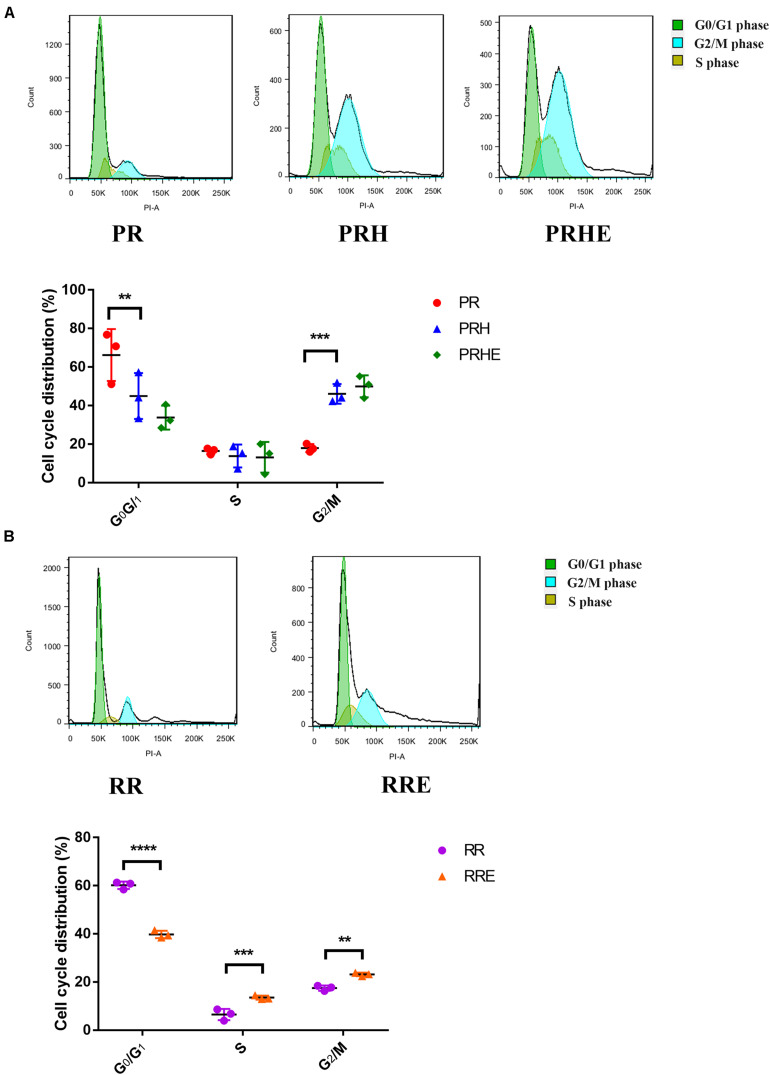
The cocultured ESCs promoted the G_1_/S phase transition in replicative senescent RPE cells. **(A)** Proportion of cell cycle distribution in the PR, PRH, and PRHE groups, as assessed by flow cytometry (*n* = 3 biological repeats). **(B)** Proportion of cell cycle distribution in the RR and RRE groups, as assessed by flow cytometry (*n* = 3 biological repeats). Data are presented as the mean ± SD. ^∗∗^*P* < 0.01; ^∗∗∗^*P* < 0.001; ^****^P < 0.0001.

Cell cycle arrest is one of the hallmarks of cellular senescence, which results in limited proliferative capacity (Nacarelli and Sell, 2017). As shown in Figure 4A, the proportion of premature senescent RPE cells in G_0_/G_1_ phase was decreased from 66.24 ± 13.46% to 44.99 ± 11.91% (*p* = 0.006), and the proportion in G_2_/M phase was increased from 17.96 ± 2.089% to 46.09 ± 5.093% (*p* = 0.0006) compared to the PR group, suggesting that H_2_O_2_-mediated premature senescence in RPE cells is mainly manifested as G_2_/M arrest, which is consistent with other studies (Santa-Gonzalez et al., 2016; Zhang et al., 2016). However, the cell cycle distribution of the PRHE group was not significantly different from that of the PRH group. In the replicative senescence model (Figure 4B), the proportion of ESCs-cocultured replicative RPE cells in G_0_/G_1_ phase was decreased from 60.19 ± 1.533% to 39.78 ± 1.545% (*p* < 0.0001) compared to that in the RR group. In particular, the proportions of RPE cells entering S phase (13.54 ± 0.8122%, *p* = 0.0002) and G_2_/M phase (23.15 ± 0.714%, *p* = 0.0013) were higher in the RRE group than those in the RR group (S phase: 6.493 ± 2.349%; G_2_/M phase: 17.48 ± 1.103%), indicating that the ESCs can enhance the proliferative capacity of replicative RPE cells mainly by promoting the cell cycle transition through a direct coculture way.

### The Cocultured ESCs Reduced Senescent Phenotypes of Premature and Replicative Senescent RPE Cells

**FIGURE 5:**
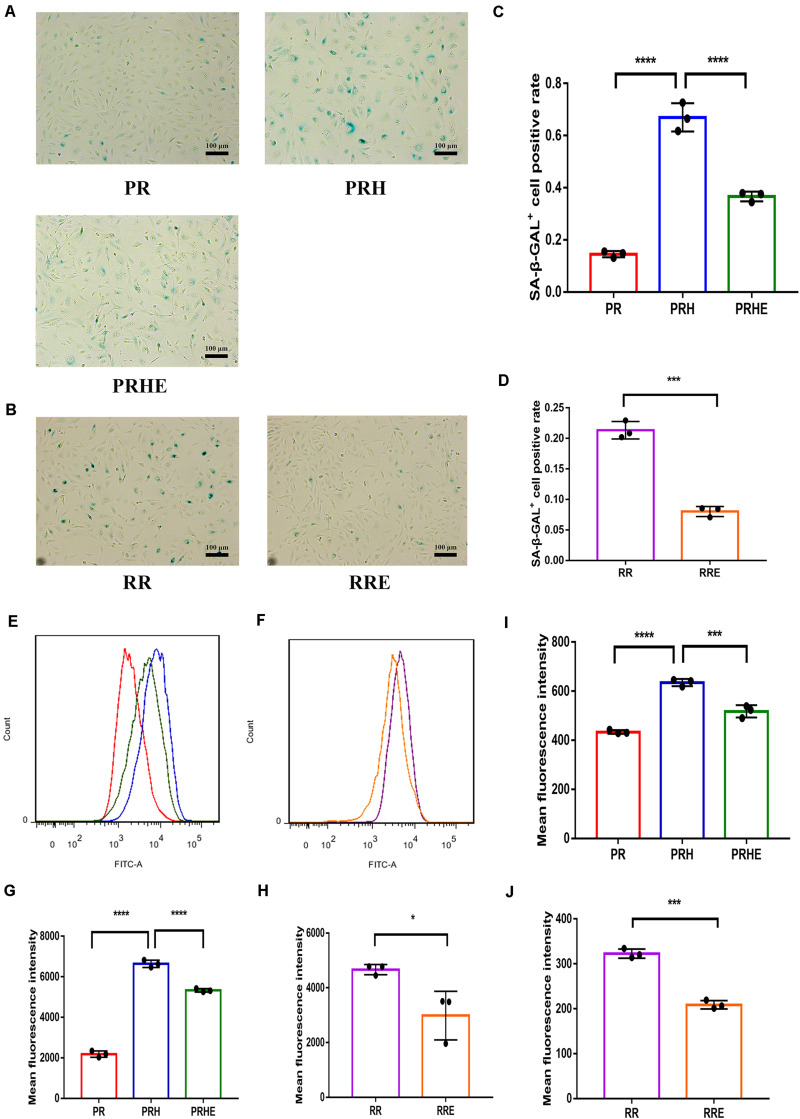
The cocultured ESCs reduced the senescent phenotypes of premature and replicative senescent RPE cells. **(A)** SA-β-GAL activity in the PR, PRH, and PRHE groups, as indicated by phase contrast microscopy. Scale bar, 100 μm. **(B)** SA-β-GAL activity in the RR and RRE groups, as indicated by phase contrast microscopy. Scale bar, 100 μm. **(C,D)** Results from the quantification of SA-β-GAL^+^ cells shown in **(A,B)**, respectively (*n* = 3 biological repeats). SA-β-GAL^+^ cells in 4 random fields were scored. The results are expressed as the percentage of stained cells. **(E)** Reactive oxygen species (ROS) levels in the PR, PRH, and PRHE groups, as assessed by flow cytometry (*n* = 3 biological repeats). **(F)** ROS levels in the RR and RRE groups, as assessed by flow cytometry (*n* = 3 biological repeats). **(G,H)** Results from the mean fluorescence intensity shown in **(E,F)**, respectively. **(I)** Mitochondrial membrane potential (MMP) levels in the PR, PRH, and PRHE groups, as assessed by a microplate reader (*n* = 3 biological repeats). **(J)** MMP levels in the RR and RRE groups, as assessed by a microplate reader (*n* = 3 biological repeats). Data are presented as the mean ± SD. ^∗^*P* < 0.05; ^∗∗∗^*P* < 0.001; ^****^P < 0.0001.

SA-β-GAL staining was observed by a light microscope. In the premature senescence model, the positive rate of SA-β-GAL staining in the PRH group was increased to 66.98 ± 5.437% compared with that in the PR group (14.48 ± 1.198%, *p* < 0.0001) (Figures 5A,C). After coculture with ESCs, the positive rate of SA-β-GAL staining in the PRHE group was decreased to 36.65 ± 1.866% (*p* < 0.0001) compared to that in the PRH group (Figures 5A,C). In the replicative senescence model, the positive rate of SA-β-GAL staining decreased from 21.33 ± 1.427% (RR group) to 8.014 ± 0.8235% (RRE group) (Figures 5B,D).

Reactive oxygen species and MMP are commonly used indicators of cellular senescence (Lee et al., 2006; Velarde et al., 2012; Banerjee and Mandal, 2015). Mean fluorescence intensity was used to indicate intracellular ROS and MMP levels. In the premature senescence model, the levels of ROS (6637 ± 177.5, *p* < 0.0001) (Figures 5E,G) and MMP (635 ± 14.36, *p* < 0.0001) (Figure 5I) in the PRH group were higher than those in the PR group. After coculture with ESCs, the levels of ROS (5329 ± 86.63, *p* < 0.0001) (Figures 5E,G) and MMP (517.8 ± 20.03, *p* = 0.003) (Figure 5I) in the PRHE group were decreased compared with those in the PRH group. In the replicative senescence model, the levels of ROS (3108 ± 673.8, *p* = 0.0229) (Figures 5F,H) and MMP (208.9 ± 9.341, *p* = 0.0001) (Figure 5J) in the RRE group were also decreased compared to those in the RR group.

### The Cocultured ESCs Downregulated Senescence-Related Positive Markers and Upregulated Senescence-Related Negative Markers

**FIGURE 6:**
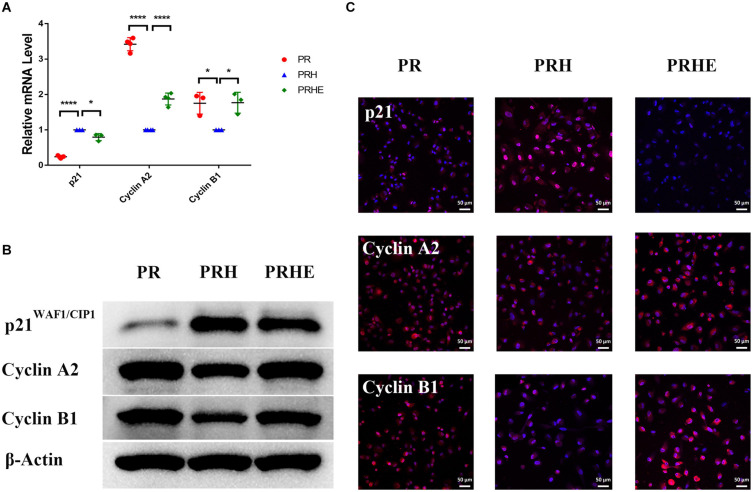
The cocultured ESCs downregulated senescence-related positive markers and upregulated senescence-related negative markers in premature senescent RPE cells. **(A)** Expression of cellular senescence-related markers in the PR, PRH, and PRHE groups, as assessed by RT- qPCR (*n* ≥ 3 biological repeats). **(B)** Results for cellular senescence-related markers in the PR, PRH, and PRHE groups as determined by western blotting. β-Actin was used as the internal reference. **(C)** The expression levels of cellular senescence-related markers in the PR, PRH, and PRHE groups as determined by immunofluorescent staining. Scale bar, 50 μm. Data are presented as the mean ± SD. ^∗^*P* < 0.05; ^****^P < 0.0001.

**FIGURE 7:**
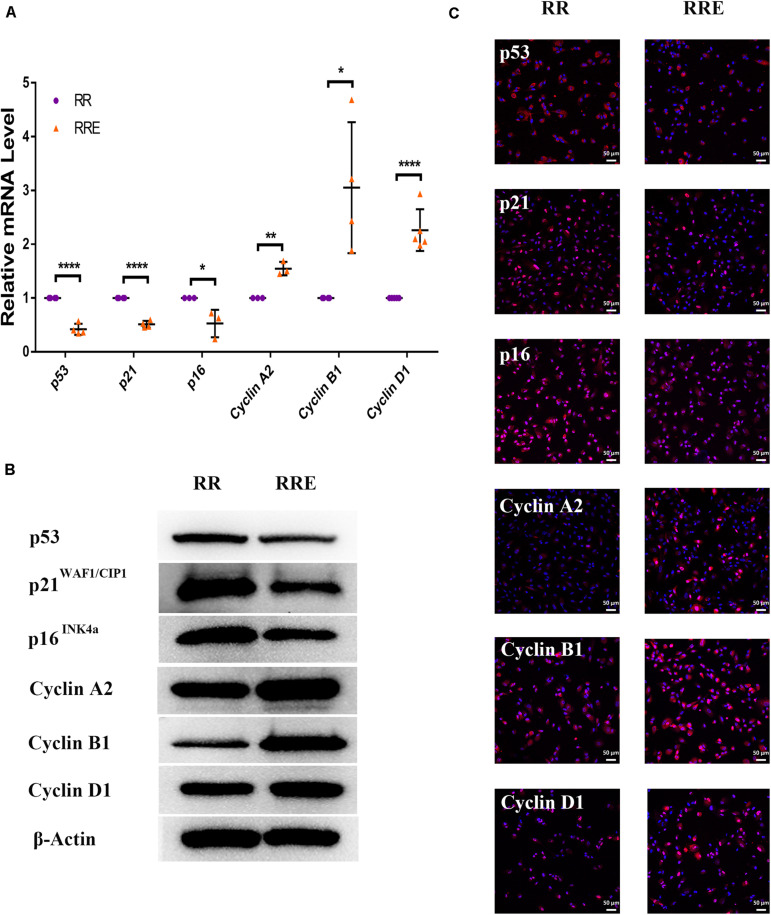
The cocultured ESCs downregulated senescence-related positive markers and upregulated senescence-related negative markers in replicative senescent RPE cells. **(A)** Expression of cellular senescence-related markers in the RR and RRE groups, as assessed by RT-qPCR (*n* ≥ 3 biological repeats). **(B)** Results for cellular senescence-related markers in the RR and RRE groups as determined by western blotting. β-Actin was used as the internal reference. **(C)** The expression levels of cellular senescence-related markers in the RR and RRE groups as determined by immunofluorescent staining. Scale bar, 50 μm. Data are presented as the mean ± SD. ^∗^*P* < 0.05; ^∗∗^*P* < 0.01; ^****^P < 0.0001.

To further verify the role of ESCs in reversing the senescence of RPE cells, we detected classical senescence-related positive (p53, p21^WAF1/CIP1^, and p16^*INK4a*^) and negative (Cyclin A2, Cyclin B1, and Cyclin D1) markers. Cyclin A2, Cyclin B1, and Cyclin D1 are cell cycle-dependent kinases that positively regulate the cell cycle (Bendris et al., 2015). As shown in Figure 6, p21^WAF1/CIP1^ was increased and Cyclin A2 and Cyclin B1 were decreased in the PRH group compared to the PR group. However, after coculture with ESCs, p21^WAF1/CIP1^ was decreased and Cyclin A2 and Cyclin B1 were increased in the PRHE group. In the replicative senescence model (Figure 7), p53, p21^WAF1/CIP1^, and p16^*INK4a*^ were downregulated, while Cyclin A2, Cyclin B1, and Cyclin D1 were upregulated in the RRE group, further suggesting that ESCs can reverse the premature and replicative senescence of RPE cells by downregulating senescence-related positive markers and upregulating senescence-related negative markers through a direct coculture way.

### The Cocultured ESCs Reversed the Premature and Replicative Senescence of RPE Cells by Regulating the TGFβ and PI3K Pathways, Respectively

**FIGURE 8:**
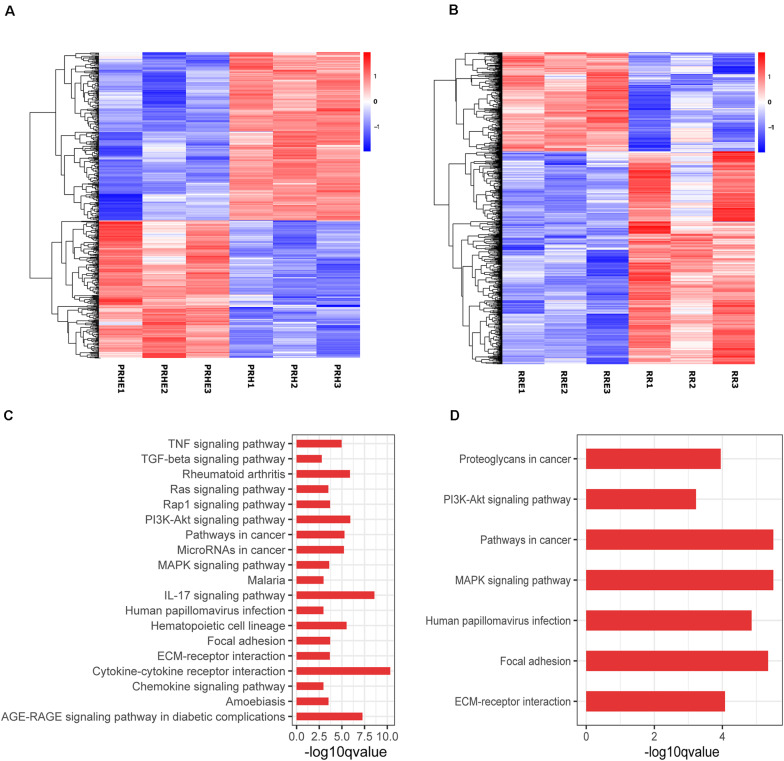
Results of RNA-seq in ESCs-cocultured premature and replicative senescent RPE cells. **(A)** The heat map of all differentially expressed genes for the PRH and PRHE groups (*n* = 3 biological repeats). **(B)** The heat map of all differentially expressed genes for the RR and RRE groups (*n* = 3 biological repeats). The horizontal axis represents three biological repeats of the samples, and the vertical axis represents the genes. The red color indicates upregulated expression and the blue color indicates downregulated expression. **(C)** The bar diagram of Kyoto Encyclopedia of Genes and Genomes (KEGG) pathway analysis of the PRH and PPHE groups (*n* = 3 biological repeats). **(D)** The bar diagram of KEGG pathway analysis of the RR and RRE groups (*n* = 3 biological repeats). The abscissa represents the enrichment degree. The greater –log10 *q* value indicates the higher enrichment degree of KEGG pathway. The ordinate represents the name of the KEGG pathway.

**FIGURE 9:**
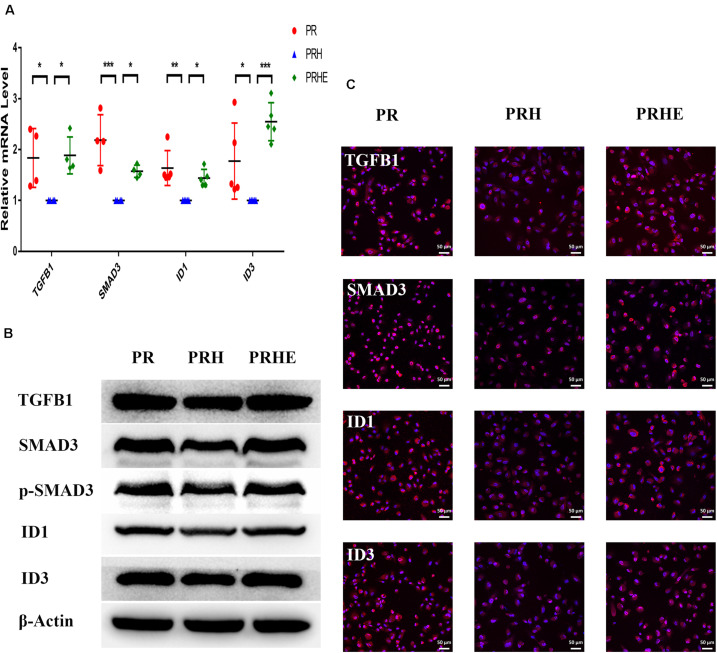
The cocultured ESCs upregulated TGFβ pathway-related markers in premature senescent RPE cells. **(A)** Expression of TGFβ pathway-related markers in the PR, PRH, and PRHE groups, as assessed by RT-qPCR (*n* ≥ 3 biological repeats). **(B)** Results for TGFβ pathway-related markers in the PR, PRH, and PRHE groups as determined by western blotting. β-Actin was used as the internal reference. **(C)** The expression levels of TGFβ pathway-related markers in the PR, PRH, and PRHE groups as determined by immunofluorescent staining. Scale bar, 50 μm. Data are presented as the mean ± SD. ^∗^*P* < 0.05; ^∗∗^*P* < 0.01; ^∗∗∗^*P* < 0.001.

**FIGURE 10:**
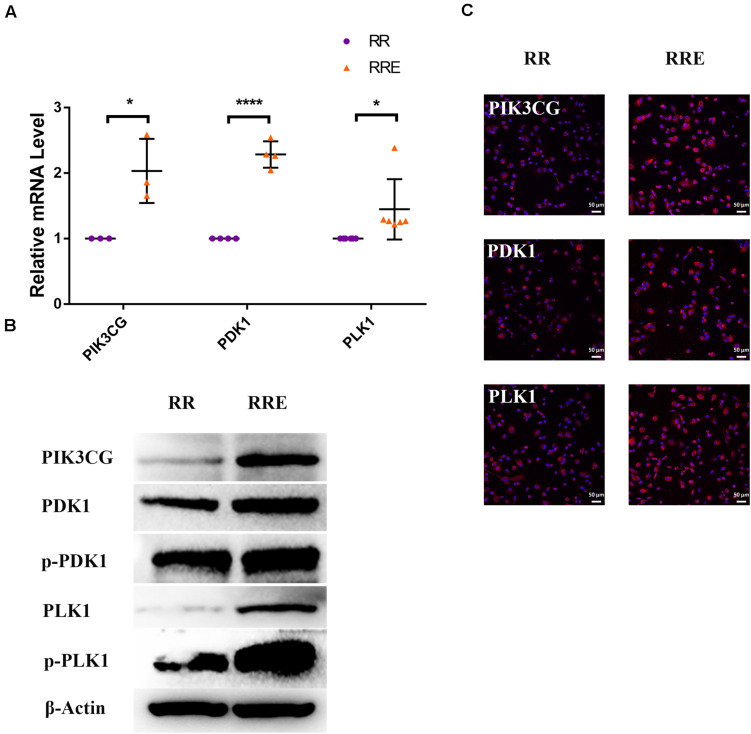
The cocultured ESCs upregulated PI3K pathway-related markers of replicative senescent RPE cells. **(A)** Expression of PI3K pathway-related markers in the RR and RRE groups, as assessed by RT-qPCR (*n* ≥ 3 biological repeats). **(B)** Results from PI3K pathway-related markers in the RR and RRE groups as determined by western blotting. β-Actin was used as the internal reference. **(C)** The expression levels of PI3K pathway-related markers in the RR and RRE groups as determined by immunofluorescent staining. Scale bar, 50 μm. Data are presented as the mean ± SD. ^∗^*P* < 0.05; ^****^P < 0.0001.

To further explore the specific mechanism by which the cocultured ESCs reversed RPE cellular senescence, RNA-seq was performed. The heat map of all differentially expressed genes (DEGs) in the premature and replicative senescent models were shown in Figures 8A,B. In order to clarify the mechanism via a whole regulatory network, we focused on the commonly used KEGG pathway analysis, which incorporates current knowledge of molecular interactive networks. The *q* value calculated by hypergeometric test was used to indicate the enrichment degree of the KEGG pathway. The smaller *q* value indicates the higher enrichment degree of KEGG pathway. In the premature senescence model, we firstly excluded KEGG pathways irrelevant to cellular senescence and RPE or KEGG pathways with enriched genes not reflecting the whole regulatory role of cellular senescence. Secondly, the representative genes in KEGG pathways that might be involved in regulating cellular senescence were in turn verified by RT-PCR or western blotting according to the *q* values from small to large, and we further excluded KEGG pathways with representative genes not significantly differentially expressed in RT-PCR. Finally, the TGFβ pathway was concerned (Figure 8C). After verified by RT-PCR, western blotting and immunofluorescence, the TGFβ pathway-related genes, including transforming growth factor beta 1 (TGFB1), SMAD family member 3 (SMAD3), inhibitor of DNA binding 1 (ID1), and inhibitor of DNA binding 3 (ID3) were decreased in the PRH group but increased in the PRHE group (Figure 9). The KEGG pathway enrichment analysis of RNA-seq data in the replicative senescence model is shown in Figure 8D. The reasons why the PI3K pathway was concerned in the replicative senescence model are similar with those of TGFβ pathway selection in the premature senescence model. The results showed that after verified by RT-PCR, western blotting and immunofluorescence, the PI3K pathway-related genes, including phosphatidylinositol-4,5-bisphosphate 3-kinase catalytic subunit gamma (PIK3CG), pyruvate dehydrogenase kinase 1 (PDK1), and polo like kinase 1 (PLK1), were increased in the RRE group compared to the RR group (Figure 10), indicating that the cocultured ESCs reversed the premature and replicative senescence of RPE cells by activating the TGFβ and PI3K pathways, respectively.

**FIGURE 11:**
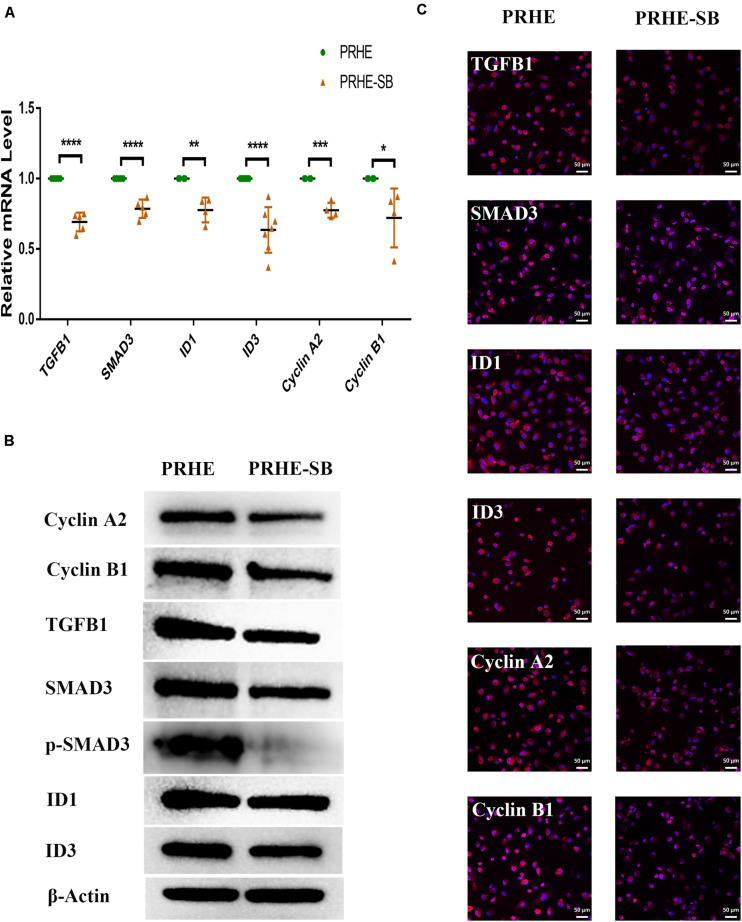
Inhibition of the TGFβ pathway upregulated senescence-related positive markers and downregulated senescence-related negative markers in ESCs-cocultured premature senescent RPE cells. **(A)** Expression of the TGFβ pathway and cellular senescence-related markers in the PRHE and PRHE-SB groups, as assessed by RT-qPCR (*n* ≥ 3 biological repeats). **(B)** Results of the TGFβ pathway and cellular senescence-related markers in the PRHE and PRHE-SB groups as determined by western blotting. β-Actin was used as the internal reference. **(C)** The expression levels of the TGFβ pathway and cellular senescence-related markers in the PRHE and PRHE-SB groups as determined by immunofluorescent staining. Scale bar, 50 μm. Data are presented as the mean ± SD. ^∗^*P* < 0.05; ^∗∗^*P* < 0.01; ^∗∗∗^*P* < 0.001; ^****^P < 0.0001. PRHE-SB: RPE cells from passages 4 to 6 treated with 400 μM H_2_O_2_ and then cocultured with ESCs with 10 μM SB431542.

**FIGURE 12:**
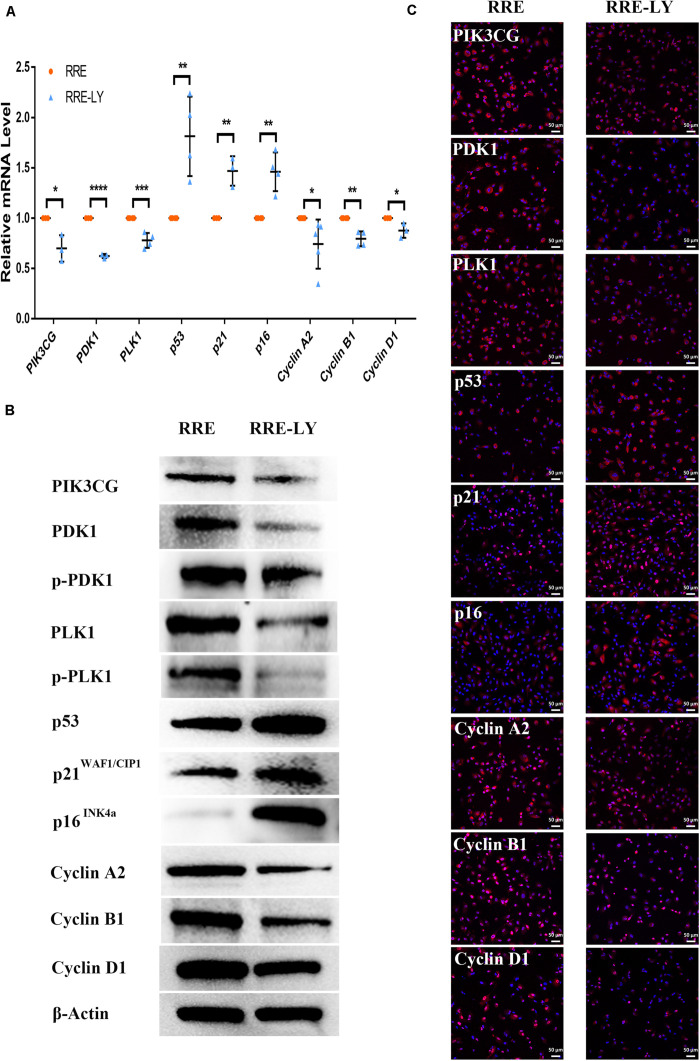
Inhibition of the PI3K pathway upregulated senescence-related positive markers and downregulated senescence-related negative markers in ESCs-cocultured replicative senescent RPE cells. **(A)** Expression of the PI3K pathway and cellular senescence-related markers in the RRE and RRE-LY groups, as assessed by RT-qPCR (*n* ≥ 3 biological repeats). **(B)** Results for the PI3K pathway and cellular senescence-related markers in the RRE and RRE-LY groups as determined by western blotting. β-Actin was used as the internal reference. **(C)** The expression levels of the PI3K pathway and cellular senescence-related markers in the RRE and RRE-LY groups as determined by immunofluorescent staining. Scale bar, 50 μm. Data are presented as the mean ± SD. ^∗^*P* < 0.05; ^∗∗^*P* < 0.01; ^∗∗∗^*P* < 0.001; ^****^P < 0.0001. RRE-LY: RPE cells from passages 8 to 10 cocultured with ESCs and treated with 10 μM LY294002.

**FIGURE 13:**
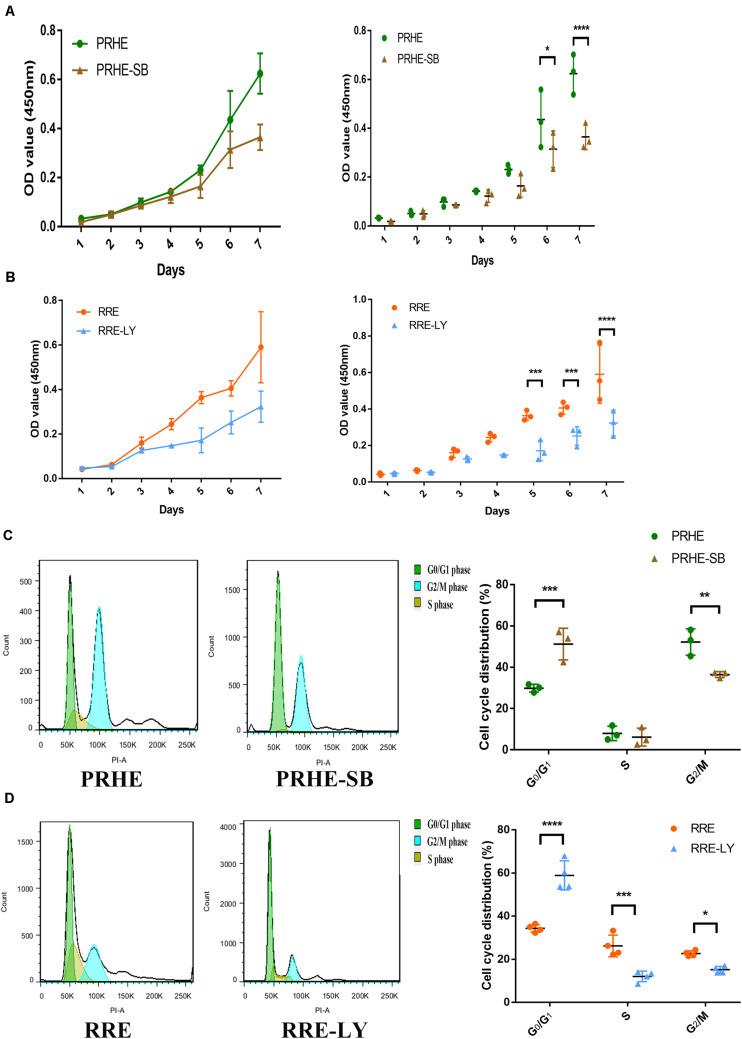
Inhibition of the TGFβ and PI3K pathways decreased the proliferative capacities and cell cycle transitions of ESCs-cocultured premature and replicative senescent RPE cells. **(A)** Proliferation of the PRHE and PRHE-SB groups, as assessed by a CCK-8 proliferation assay (*n* = 3 biological repeats). **(B)** Proliferation of the RRE and RRE-LY groups, as assessed by a CCK-8 proliferation assay (*n* = 3 biological repeats). **(C)** Proportion of cell cycle distribution in the PRHE and PRHE-SB groups, as assessed by flow cytometry (*n* = 3 biological repeats). **(D)** Proportion of cell cycle distribution in the RRE and RRE-LY groups, as assessed by flow cytometry (*n* = 4 biological repeats). Data are presented as the mean ± SD. ^∗^*P* < 0.05; ^∗∗^*P* < 0.01; ^∗∗∗^*P* < 0.001; ^****^P < 0.0001.

**FIGURE 14:**
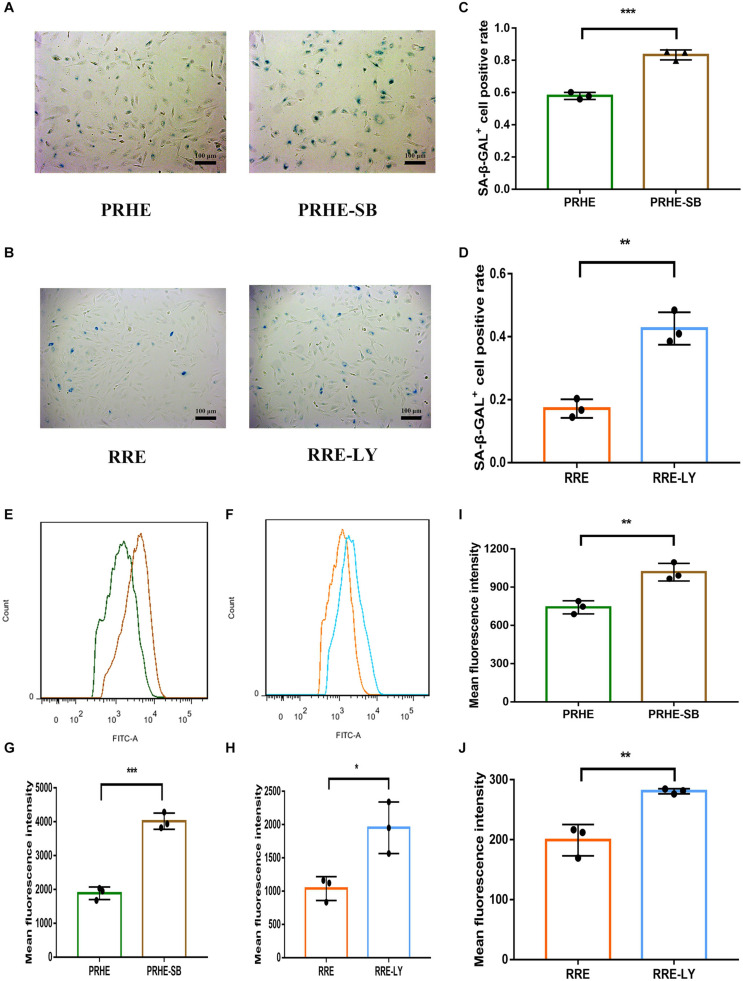
Inhibition of the TGFβ and PI3K pathways increased the senescent phenotype of ESCs-cocultured premature and replicative senescent RPE cells. **(A)** SA-β-GAL activity in the PRHE and PRHE-SB groups, as indicated by phase contrast microscopy. Scale bar, 100 μm. **(B)** SA-β-GAL activity in the RRE and RRE-LY groups, as indicated by phase contrast microscopy. Scale bar, 100 μm. **(C,D)** Results from the quantification of SA-β-GAL^+^ cells shown in **(A,B)**, respectively (*n* = 3 biological repeats). SA-β-GAL^+^ cells in 4 random fields were scored. The results are expressed as the percentage of stained cells. **(E)** ROS levels in the PRHE and PRHE-SB groups, as assessed by flow cytometry (*n* = 3 biological repeats). **(F)** ROS levels in the RRE and RRE-LY groups, as assessed by flow cytometry (*n* = 3 biological repeats). **(G,H)** Results from the mean fluorescence intensity shown in **(E,F)**, respectively. **(I)** MMP levels in the PRHE and PRHE-SB groups, as assessed by a microplate reader (*n* = 3 biological repeats). **(J)** MMP levels in the RRE and RRE-LY groups, as assessed by a microplate reader (*n* = 3 biological repeats). Data are presented as the mean ± SD. ^∗^*P* < 0.05; ^∗∗^*P* < 0.01; ^∗∗∗^*P* < 0.001.

Furthermore, SB-431542 (Scharpfenecker et al., 2009), a specific inhibitor of the TGFβ pathway, and LY294002 (Qin et al., 2013), a specific inhibitor of the PI3K pathway, were used in ESCs-cocultured premature and replicative senescent PRE cells. The results showed that the expression levels of TGFB1, SMAD3, ID1, ID3, Cyclin A2, and Cyclin B1 in the PRHE-SB group were reduced compared with those in the PRHE group (Figure 11). Similarly, the expression of PIK3CG, PDK1, PLK1, Cyclin A2, Cyclin B1, and Cyclin D1 in the RRE-LY group was decreased, while p53, p21, and p16 levels were increased compared with those in the RRE group (Figure 12). Moreover, the proliferative capacity of the PRHE-SB group was decreased (Figure 13A), while the positive rate of SA-β-GAL staining (83.37 ± 3.065%, *p* = 0.0003) and the levels of ROS (4016 ± 240, *p* = 0.0003) and MMP (1017 ± 68.6, *p* = 0.0051) were increased compared with those in the PRHE group (SA-β-GAL^+^:57.94 ± 2.197%; ROS:1888 ± 185.3; MMP:742 ± 51.21) (Figures 14A,C,E,G,I). The proportion of PRHE-SB group in G_0_/G_1_ phase was increased from 29.87 ± 1.929% to 51.25 ± 7.681% (*p* = 0.0004), and the proportion in G_2_/M phase was decreased from 52.21 ± 6.388% to 36.45 ± 1.415% (*p* = 0.0048) compared to the PRHE group (Figure 13C). Similarly, compared to the RRE group (SA-β-GAL^+^:17.17 ± 2.965%; ROS:1039 ± 178.9; MMP:199.3 ± 26.09), the proliferative capacity of RRE-LY group was decreased (Figure 13B), while the positive rate of SA-β-GAL staining (42.63 ± 5.149%, *p* = 0.0018) and the levels of ROS (1953 ± 388, *p* = 0.0208) and MMP (280.9 ± 4.221, *p* = 0.0059) were elevated (Figures 14B,D,F,H,J). The proportion of RRE-LY group in G_0_/G_1_ phase was increased from 34.33 ± 1.773% to 58.88 ± 6.783% (*p* < 0.0001), and the proportions of RPE cells entering S phase (12.01 ± 2.434%, *p* = 0.0001) and G_2_/M phase (15.14 ± 1.554%, *p* = 0.0337) were lower than those in the RRE group (S phase: 26.17 ± 5.011%; G_2_/M phase: 22.61 ± 1.286%) (Figure 13D). All of these results suggest that after the application of the corresponding inhibitors, the TGFβ pathway and PI3K pathway in ESCs-cocultured premature and replicative senescent PRE cells were inhibited, resulting in the downregulation of senescence-related positive markers, proliferation and cell cycle transition, and the upregulation of senescence-related negative markers, SA-β-GAL staining positive rate and levels of ROS and MMP, further suggesting that the cocultured ESCs reversed the premature and replicative senescence of RPE cells possibly by regulating the TGFβ and PI3K pathways, respectively.

## Discussion

Currently, stem cell-induced differentiation and transplantation are the main anti-aging methods for treating age-related diseases (da Costa et al., 2016). Although stem cells can be used to differentiate into functional RPE cells, there are some problems, such as low differentiation efficiency, tumorigenicity and unknown safety issues (Mandai et al., 2017), which limits their clinical application. RPE cellular senescence is one of the important causes of AMD (Wang et al., 2019). Although senescence in RPE cells can be delayed by antioxidant drugs (Zhuge et al., 2014; Sreekumar et al., 2016), almost all drugs have off-target and bystander effects (Wang et al., 2019), and senescent cells cannot be cleared by delaying cellular senescence. Therefore, reversal of RPE cellular senescence may be an effective treatment for AMD.

Recently, utilizing a young environment as anti-aging treatment has been a research hotspot. For example, the plasma of young mice rejuvenated older mice (Rebo et al., 2016). Our previous studies found that ESC-conditioned medium promoted the proliferation of corneal epithelial and endothelial cells and increased the expression of stem cell markers in those cells (Liu et al., 2010; Lu et al., 2010). Furthermore, direct coculture had a stronger effect than indirect coculture assessed by the transwell assay and ESC-conditioned medium (Zhou et al., 2011). We also demonstrated that ESCs can reverse the malignancy of leukemia and choroidal melanoma by a direct coculture way and promote the proliferation of normal skin tissues adjacent to tumors. However, the microenvironment of mesenchymal stem cells did not have this effect (Zhou et al., 2014; Liu et al., 2019). On the basis of our previous studies, we used ESCs to directly coculture with RPE cells.

SA-β-GAL staining, ROS levels and MMP levels are the most commonly used markers of cellular senescence (Aravinthan, 2015; Jing et al., 2018). The nuclei of senescent cells can be stained blue with SA-β-GAL, which is a gold standard of cellular senescence detection (de Mera-Rodriguez et al., 2019). Oxidative stress often leads to increased intracellular ROS, and long-term accumulation of ROS is considered to be a driver of senescence (Velarde et al., 2012). If oxidative stress does not cause mitochondrial dysfunction but mitochondrial hyperpolarization, it leads to elevated MMP (Lee et al., 2006). Elevated MMP leads to an increase in ROS (Zorov et al., 2006), thus aggravating oxidative stress damage and cellular senescence. In this study, after coculture with ESCs, the positive rate of SA-β-GAL staining and the levels of ROS and MMP were decreased (Figure 5), which fully demonstrated the potential of cocultured ESCs to reverse the senescence of RPE cells.

Typical cellular senescence is characterized by cell cycle arrest, decreased self-renewal and repair abilities, and restricted growth (Nacarelli and Sell, 2017). Cyclin A2, Cyclin B1, and Cyclin D1 are classical markers of the cell cycle. The expression of Cyclin A2 increases at the transition of G_1_ to S phase and continues to G_2_ phase. Cyclin B1 promotes the G_2_/M phase transition, and Cyclin D1 promotes the G_1_/S phase transition (Oredsson, 2003). P53, p21^WAF1/CIP1^, and p16^*INK4a*^ are classical positive markers of cellular senescence (Sikora et al., 2016) and inhibit a series of cyclins after activation (Campisi and Robert, 2014; Mowla et al., 2014). In this study, after coculture with ESCs, the proliferative capacity of premature and replicative senescent RPE cells was significantly improved (Figure 3). Moreover, replicative RPE cells entered the S and G_2_/M phases (Figure 4B). Positive markers of cellular senescence (premature senescent RPE: p21; replicative senescent RPE: p53, p21, and p16) were downregulated, while negative markers (premature senescent RPE: Cyclin A2 and Cyclin B1; replicative senescent RPE: Cyclin A2, Cyclin B1, and Cyclin D1) were upregulated (Figures 6,7), suggesting that the cocultured ESCs reversed the senescence of RPE cells by promoting cell cycle transition through p53, p21^WAF1/CIP1^, and p16^*INK4a*^. However, there was no significant difference in the cell cycle distribution of ESCs-cocultured premature senescent RPE cells (Figure 4A). Combined with other results, we observed the effect of cocultured ESCs reversing premature senescence of RPE cells.

ID family genes, downstream of the TGFβ pathway, participate in cell cycle regulation. ID proteins can downregulate p21^WAF1/CIP1^ and activate cyclin-dependent protein kinases (CDKs) by antagonizing class A and class B heterodimers, thereby promoting cell cycle transition. The overexpression of ID1 and ID3 can delay the senescence of human keratinocytes (Zebedee and Hara, 2001). TGFB1 can mediate ID expression through SMAD3 (Liang et al., 2009; Notohamiprodjo et al., 2012). In this study, we found that TGFB1, SMAD3, ID1, and ID3 were upregulated in ESCs-cocultured premature senescent RPE cells, accompanied by the downregulation of p21^WAF1/CIP1^ and the upregulation of Cyclin A2 and Cyclin B1 (Figures 8A, 9). After SB431542 was applied, TGFB1, SMAD3, ID1, and ID3 were decreased while Cyclin A2 and Cyclin B1 were increased (Figure 11) with the deceased proliferation and cell cycle transition (Figures 13A,C) and the increased positive rate of SA-β-GAL staining and levels of ROS and MMP (Figures 14A,C,E), suggesting that the cocultured ESCs reversed the premature senescence of RPE cells possibly by activating the TGFβ pathway, which then upregulated Cyclin A2 and Cyclin B1 and downregulated p21^WAF1/CIP1^.

Studies have shown that the activation of the PI3K pathway can delay cellular senescence (Chen et al., 2017; Chai et al., 2018; Wang et al., 2018). With increasing age, the activity of the PI3K pathway is decreased, resulting in reduced tolerance to stress-induced mitochondria and cell damage, while the activation of the PI3K pathway contributes to the recovery of the functions of senescent RPE cells (He et al., 2014), indicating that the PI3K pathway is involved in the regulation of RPE cellular senescence. PI3K can directly activate PDK1 to promote cell proliferation independently of AKT (Xia et al., 2018). PDK1 can directly mediate PLK1 phosphorylation (Tan et al., 2013). PLK1 is involved in many mitotic processes, and the upregulation of PLK1 can reverse part of the aging phenotype (Kim et al., 2013). Second, PLK1 can directly bind to p53 to antagonize its function (Shao et al., 2018). The expression of p21^WAF1/CIP1^ is increased after PLK1 is knocked out (Zhang et al., 2015). The results of this study showed that the expression of PIK3CG, PDK1, and PLK1 was increased in ESCs-cocultured replicative senescent RPE cells (Figure 10). After LY294002 was applied, PIK3CG, PDK1, PLK1, Cyclin A2, Cyclin B1, and Cyclin D1 were decreased while p53, p21^WAF1/CIP1^, and p16^*INK4a*^ were increased (Figure 12) with the deceased proliferation and cell cycle transition (Figures 13B,D) and the increased positive rate of SA-β-GAL staining and levels of ROS and MMP (Figures 14B,D,F), suggesting that the cocultured ESCs may reverse the replicative senescence of RPE cells by activating the PI3K pathway, further downregulating p53, p21^WAF1/CIP1^, and p16^*INK4a*^ and upregulating Cyclin A2, Cyclin B1, and Cyclin D1.

In summary, this study demonstrates for the first time that the ESCs can reverse the premature and replicative senescence of RPE cells by a direct coculture way, which may be achieved by upregulating the TGFβ and PI3K pathways, respectively, providing a new option for stem cell-based therapy of AMD and for anti-aging treatment via a young environment in the future.

## Data Availability Statement

The RNA-seq data used in this study is publicly available in the Sequence Read Archive (SRA) database with the accession number PRJNA671771. Other raw data supporting the conclusions of this article will be made available by the authors on reasonable request, without undue reservation.

## Ethics Statement

The studies involving human participants were reviewed and approved by the Ethics Committee of Zhongshan Ophthalmic Center, Sun Yat-sen University. The patients/participants provided their written informed consent to participate in this study. Written informed consent was obtained from the individual(s) for the publication of any potentially identifiable images or data included in this article.

## Author Contributions

SW conceived the concept, conducted the experiments, and wrote the manuscript. YRL conducted the experiments, interpreted the results, and edited the manuscript. YL, CYL, and LY conducted the experiments. QW prepared the figures. YS, YC, and CL supervised the study. XW and ZW conceived the concept and edited the manuscript. All authors approved the manuscript.

## Conflict of Interest

The authors declare that the research was conducted in the absence of any commercial or financial relationships that could be construed as a potential conflict of interest.
