# Supplementary material for: Reversed Senescence of Retinal Pigment Epithelial Cell by Coculture With Embryonic Stem Cell via the TGFβ and PI3K Pathways
Source: Front Cell Dev Biol. 2020 Nov 26;8:588050. doi: 10.3389/fcell.2020.588050 (PMC7726211; doi:10.3389/fcell.2020.588050)
Supplement: Supplementary file 1 [file Data_Sheet_1.docx]

Supplementary Material


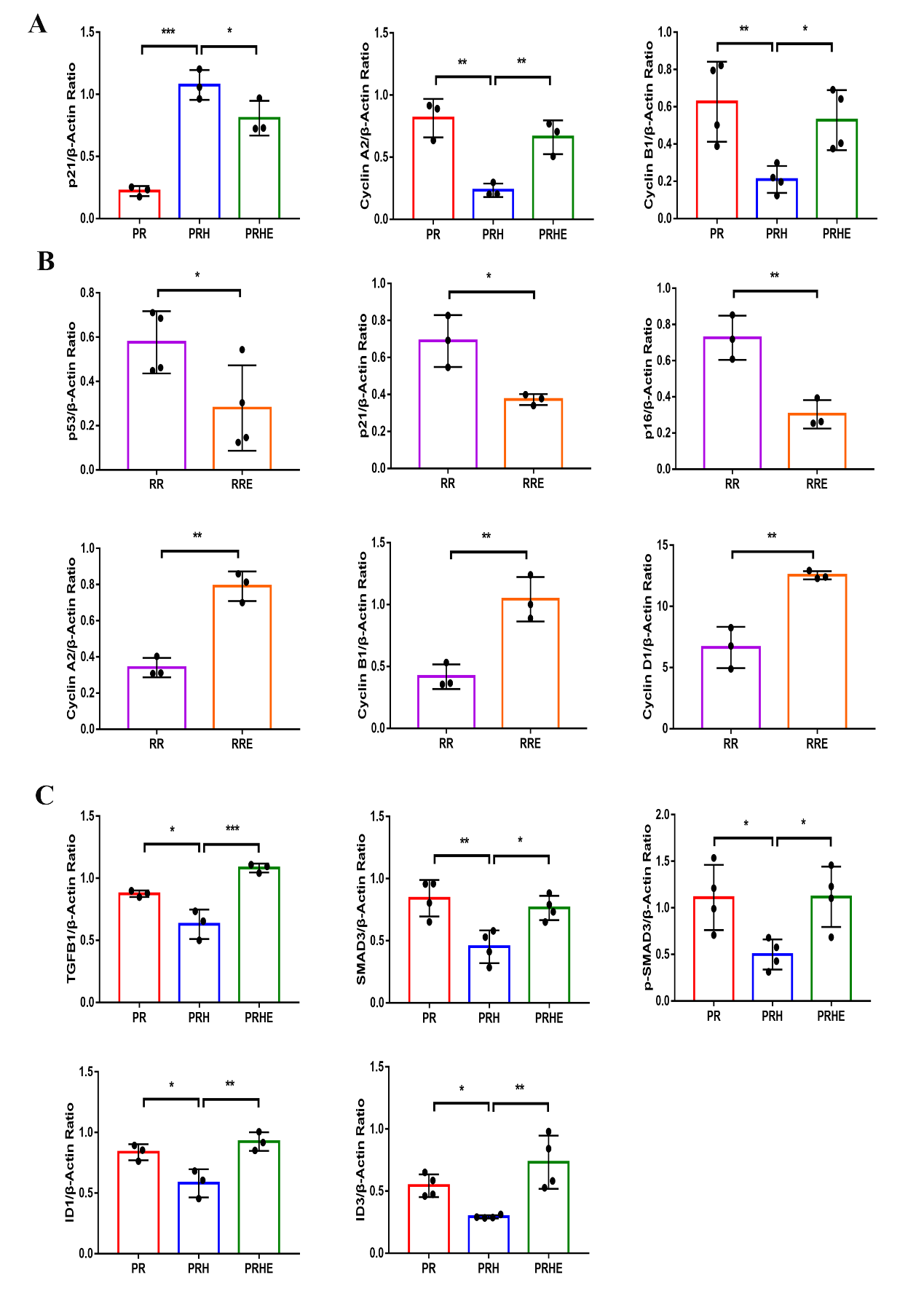


**Supplementary Figure 1. Quantification of immunoblots.** (A) Quantifications of p21, Cyclin A2 and Cyclin B1 in Figure 6B (n≥3 biological repeats). (B) Quantifications of p53, p21, p16, Cyclin A2, Cyclin B1 and Cyclin D1 in Figure 7B (n≥3 biological repeats); (C) Quantifications of TGFB1, SMAD3, p-SMAD3, ID1 and ID3 in Figure 9B (n≥3 biological repeats). Data are presented as the mean±SD. *P<0.05; **P<0.01; ***P<0.001.

**Supplementary Table 1.** Antibodies used in Western blotting (WB) and Immunofluorescence (IF).

| Antibody | Source | Dilution (Application) |
| --- | --- | --- |
| Anti-p53 | Abcam#ab32389 | 1:1000 (WB)  1:50 (IF) |
| Anti-p21^WAF1/CIP1^ | Abcam#ab109520 | 1:1000 (WB)  1:1000 (IF) |
| Anti-p16^INK4a^ | Abcam#ab108349 | 1:2000 (WB)  1:270 (IF) |
| Anti-Cyclin A2 | Abcam#ab32386 | 1:10000 (WB)  1:100 (IF) |
| Anti-Cyclin B1 | Abcam#ab181593 | 1:2000 (WB)  1:500 (IF) |
| Anti-Cyclin D1 | Abcam#ab134175 | 1:10000 (WB)  1:50 (IF) |
| Anti-TGF-β1 | Abcam#ab27969 | 1:2000 (WB)  1:150 (IF) |
| Anti-SMAD3 | Abcam#ab40854 | 1:1000 (WB)  1:500 (IF) |
| Anti-pSMAD3 | Abcam#ab52903 | 1:2000 (WB) |
| Anti-ID1 | Abcam#ab168256 | 1μg/ml (WB)  10μg/ml (IF) |
| Anti-ID3 | Abcam#ab236505 | 1:500 (WB)  1:100 (IF) |
| Anti-PIK3CG | Abcam#ab238509 | 1:500 (WB)  1:50 (IF) |
| Anti-PDK1 | Abcam#ab110025 | 1:500 (WB)  1:200 (IF) |
| Anti-pPDK1 | Cell Signaling Technology#3061 | 1:1000 (WB) |
| Anti-PLK1 | Abcam#ab17057 | 1μg/ml (WB)  1:200 (IF) |
| Anti-pPLK1 | Abcam#ab39068 | 1μg/ml (WB) |
| Anti-PDGFRβ | Abcam#ab32570 | 1:5000 (WB) |
| Anti-CD31 | Abcam#ab76533 | 1:5000 (WB) |
